# Genome-Wide DNA Methylation and Transcriptome Integration Associates DNA Methylation Changes with Bovine Subclinical Mastitis Caused by *Staphylococcus chromogenes*

**DOI:** 10.3390/ijms241210369

**Published:** 2023-06-20

**Authors:** Mengqi Wang, Nathalie Bissonnette, Mario Laterrière, David Gagné, Pier-Luc Dudemaine, Jean-Philippe Roy, Marc-André Sirard, Eveline M. Ibeagha-Awemu

**Affiliations:** 1 Sherbrooke Research and Development Centre, Agriculture and Agri-Food Canada, Sherbrooke, QC J1M 0C8, Canada; mengqi.wang@agr.gc.ca (M.W.); nathalie.bissonnette@agr.gc.ca (N.B.); pier-luc.dudemaine@agr.gc.ca (P.-L.D.); 2 Department of Animal Science, Université Laval, Québec, QC G1V 0A6, Canada; marc-andre.sirard@fsaa.ulaval.ca; 3 Quebec Research and Development Centre, Agriculture and Agri-Food Canada, Quebec, QC G1V 2J3, Canada; 4 Department of Clinical Sciences, Université de Montréal, Saint-Hyacinthe, QC H3T 1J4, Canada; jean-philippe.roy@umontreal.ca

**Keywords:** differential methylation haplotype blocks (dMHBs), differentially methylated and expressed genes (DMEGs), negative association, promoter, first exon, first intron, immune functions, discriminant signatures

## Abstract

*Staphylococcus chromogenes* (SC) is a common coagulase-negative staphylococcus described as an emerging mastitis pathogen and commonly found in dairy farms. This study investigated the potential involvement of DNA methylation in subclinical mastitis caused by SC. The whole-genome DNA methylation patterns and transcriptome profiles of milk somatic cells from four cows with naturally occurring SC subclinical mastitis (SCM) and four healthy cows were characterized by next-generation sequencing, bioinformatics, and integration analyses. Comparisons revealed abundant DNA methylation changes related to SCM, including differentially methylated cytosine sites (DMCs, *n* = 2,163,976), regions (DMRs, *n* = 58,965), and methylation haplotype blocks (dMHBs, *n* = 53,098). Integration of methylome and transcriptome data indicated a negative global association between DNA methylation at regulatory regions (promoters, first exons, and first introns) and gene expression. A total of 1486 genes with significant changes in the methylation levels of their regulatory regions and corresponding gene expression showed significant enrichment in biological processes and pathways related to immune functions. Sixteen dMHBs were identified as candidate discriminant signatures, and validation of two signatures in more samples further revealed the association of dMHBs with mammary gland health and production. This study demonstrated abundant DNA methylation changes with possible involvement in regulating host responses and potential as biomarkers for SCM.

## 1. Introduction

Mastitis is a common and costly disease of dairy cows that causes inflammation resulting in decreased milk quality and quantity. Mastitis is caused by a plethora of pathogens, including *Staphylococcus chromogenes* (SC). *Staphylococcus chromogenes* is one of the most predominant species of coagulase-negative staphylococci (CNS) and is considered an emerging mastitis pathogen of dairy cows in many countries [1,2]. It is also the most predominant CNS on Canadian dairy farms [3,4,5]. This pathogen can cause persistent and recurring infections, leading to chronic mastitis, which can have significant negative impacts on dairy cow welfare and farm incomes [6,7,8]. Therefore, a better understanding of the mechanisms of bovine mastitis caused by SC is crucial for the development of effective control measures to improve the health and welfare of dairy cows and to ensure the sustainability of the dairy industry.

In time past, CNS were believed to have low pathogenic potential, generally regarded as saprophytic microflora and classified as ‘secondary bacteria’ [9]. In recent years, however, CNS are among the predominant pathogens isolated from dairy farms with an incidence of subclinical mastitis in many countries, consequently attracting research attention [1,10]. In order to better understand and control the mastitis caused by CNS, some studies have focused on the genetic factors that contribute to the pathogenesis of bovine CNS mastitis. These studies characterized the molecular mechanisms underlying bacterial virulence and identified potential targets for the development of new treatments and control strategies [11,12,13]. For example, genetic characterization of CNS identified virulence factors and multidrug resistance strains, which informed the adoption of appropriate hygienic measures and treatment strategies to prevent the development of antibiotics resistance due to CNS bacteria [14,15,16]. However, the genetic/epigenetic regulation of host responses to mastitis caused by CNS bacteria is scarcely reported in dairy cows, which leads to a research gap in genetic selection for improving mastitis resistance.

With recent developments in the subject area of epigenetics, epigenetic modifications have been revealed to play important roles in the health and productivity of livestock [17,18,19]. DNA methylation, as the most studied epigenetic mechanism, has been shown to modulate genome function and play crucial roles in the regulation of cellular processes [20,21]. In the context of bovine mastitis, DNA methylation has been identified as an important factor in the regulation of the host immune responses [22,23,24]. By regulating the expression of specific genes, DNA methylation could affect the ability of pathogens to colonize and cause infection in the mammary gland, as well as the host’s ability to defend itself against invading pathogens [25,26,27]. Furthermore, understanding the role of DNA methylation in mastitis pathogenesis may provide insights into the molecular mechanisms that underlie bacterial adaptation to the host environment. DNA methylation has been shown to play a role in the regulation of genes involved in bacterial adherence, biofilm formation, antibiotic resistance, and other virulence factors [28,29,30,31,32]. Therefore, investigating the epigenetic changes that underlie the host response to bacterial infections may provide new insights into the mechanisms involved in the host–pathogen interaction in bovine mastitis.

Unfortunately, limited studies have explored the DNA methylation changes in immune-related genes during mastitis caused by CNS [25,33]. For instance, hyper-methylation of the upstream elements of *TERT* was detected to be associated with its down-regulated expression during CNS infection [33]. However, the involvement of DNA methylation in bovine mastitis caused by SC is not known. Therefore, the study of DNA methylation in relation to SC mastitis continues to be an important area of research, with the potential to contribute to improving our understanding of the molecular basis of mastitis pathogenesis.

Therefore, we profiled the whole-genome DNA methylation patterns and mRNA transcriptome of milk somatic cells from dairy cows with naturally occurring SC subclinical mastitis using whole-genome DNA methylation sequencing (WGMS) and RNA sequencing. Then, we performed comparative analyses to investigate the DNA methylation changes relevant to SC subclinical mastitis. In addition, we integrated the DNA methylation profile with the mRNA transcriptome profile to understand the association between DNA methylation and gene expression activities during SC subclinical mastitis and explore their possible regulatory roles in host responses to SC invasion. Furthermore, we also identified candidate discriminant signatures for SC subclinical mastitis and validated two DNA methylation signatures in a larger sample size to further reveal their association with mammary gland health and milk production traits. In summary, this study revealed the possible involvement of DNA methylation changes in SC subclinical mastitis, which may serve as a reference for further research on the potential roles of DNA methylation in bovine subclinical mastitis caused by CNS.

## 2. Results

### 2.1. Methylome Profiling Revealed Abundant DNA Methylation Alterations during SC Subclinical Mastitis

**Figure 1:**
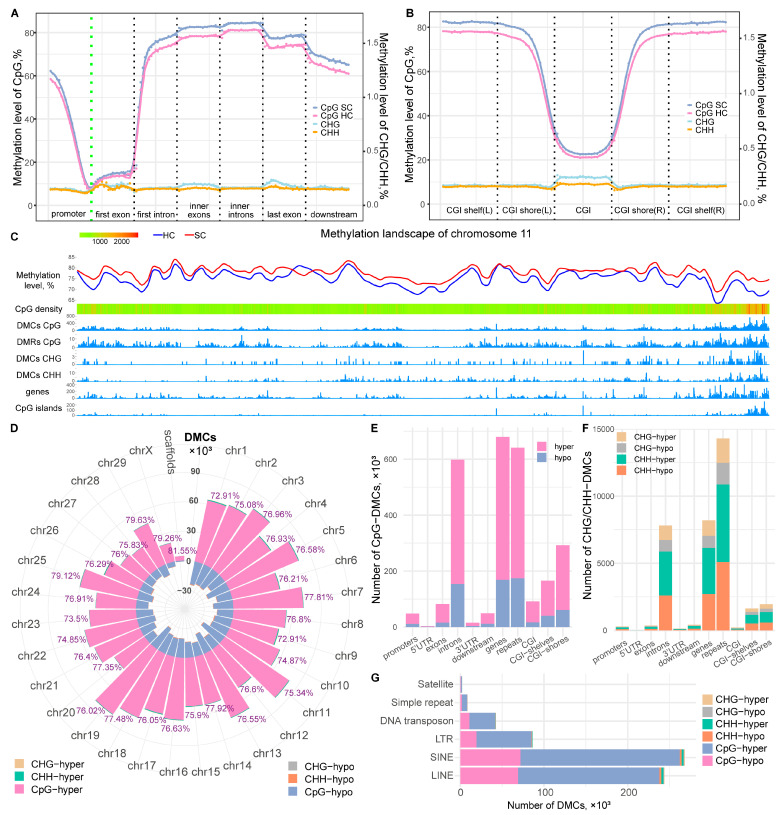
DNA methylation landscape of milk somatic cells and the identification of DNA methylation changes. (**A**) The global DNA methylation trends among genic structures; (**B**) The global DNA methylation trends around CpG islands (CGI); (**C**) The DNA methylation landscape of chromosome 11. The various tracks show DNA methylation changes, genes, and CpG islands, which were counted using a 50 kb window and displayed in a line plot (first track for methylation level), density distribution map (second track for CpG density), or histogram plot (other tracks); (**D**) The distribution of differentially methylated cytosine sites (DMCs) among chromosomes (chr). The number on the top bar represents the percentage of hyper-methylated DMCs to total DMCs in the corresponding chr; (**E**) The distribution of CpG-DMCs in genic structures and genomic regions; (**F**) The distribution of DMCs in the context of CHG and CHH in genic structures and genomic regions; (**G**) The distribution of DMCs in repeat elements. LTR: long terminal repeat retrotransposon, SINE: short interspersed nuclear elements, LINE: long interspersed nuclear elements. More details on data shown in (**D**–**G**) are found in Supplementary Table S5.

In this study, we generated eight WGMS datasets of high quality for milk somatic cells from four cows positive for SC-subclinical mastitis (SCP) and four healthy control cows (HC). Each WGMS dataset contained about 475 million reads ranging from 450 to 550 million, with an average read coverage depth of ~27× (Table S1). After quality control (adapter sequences and poor quality reads removed), about 353 million reads per sample were aligned to the bovine reference genome (ARS-UCD1.2), and ~85% of them were uniquely aligned. Finally, we identified about 12.3 billion cytosine sites in 3 contexts- CpG, CHG, or CHH (where H stands for A or T or C). The CpG sites showed a 73.68% methylation level in milk somatic cells, which is similar to other bovine somatic tissues [34]. Meanwhile, the CHG and CHH sites showed extremely low methylation levels (0.2% and 0.16%, respectively). The methylation level trend among genic structures revealed the classic loss of CpG methylation around transcript start sites (TSSs) as well as CpG islands (CGI) (Figure 1A,B). It should be noted that TSSs are often accompanied by CGIs in mammals. The first exons had significantly lower CpG methylation levels (<20%) than other regions. The CpG methylation level showed a gradual upward increase at the first introns, which reached maximum high levels at inner exons and introns, followed by a slight decline at the last exon and downstream region (Figure 1A). On the contrary, CHG and CHH methylation showed slightly higher levels at CGI (Figure 1B).

A comparison of DNA methylation profiles between SCP and HC groups revealed abundant differences. First, the global methylation level of CpG sites was significantly higher in the SCP group compared to the HC group in genic regions (Figure 1A,B and Table S2) and chromosomes (Figure 1C showing chromosome 11 as an example). Second, we identified about 2.17 million differentially methylated cytosine sites (DMCs) between SCP and HC, and more than 98% of them were CpG-DMCs (Table S3). Only 7494 and 22,860 DMCs were identified in the context of CHG and CHH, respectively (Table S4A,B). Consistent with the global higher methylation level in the SCP group, about 75% of CpG-DMCs and 50% of CHG-/CHH-DMCs were hyper-methylated in the SCP group (Table S5). Chromosomes 19, 11, and 5 are the top 3 chromosomes that harbored the most DMCs (more than 10 thousand DMCs) (Figure 1D and Table S5A). Moreover, the annotation of DMCs to genic structures indicated that the majority of DMCs (68%) are located in the intergenic regions (Table S5B). Among DMCs collocated with genic structures, introns harbored the most DMCs, followed by exons, downstream, and promoter regions (Figure 1E,F). Considering location in relation to CGI, most DMCs were found in CGI deserts, while CGI shores harbored more DMCs than CGI and CHI shelves (Figure 1E,F). Moreover, a high number of DMCs were also collocated with repeat elements, mostly short and long interspersed nuclear elements (SINE, LINE) and long terminal repeats retrotransposons (LTRs) (Figure 1G). In addition, we also identified a total of 58,965 differentially methylated regions (DMRs) based on CpGs by using a window size of 1 kb. About 80% of DMRs had higher methylation levels in the SCP group. DMRs showed a similar distribution with CpG-DMCs among genic structures and genomic regions (Table S6).

Interestingly, and as depicted in the DNA methylation landscape of chromosome 11 (Figure 1C), peak DNA methylation changes, including DMCs and DMRs, were mostly accompanied by peak gene density. Therefore, we further checked the distribution of DMCs within individual genes. We found a total of 13,743 genes having CpG-DMCs in their gene body (including promoter and downstream region), 78.62% of which harbored ≥10 CpG-DMCs (Table S7A). Fewer genes (*n* = 1360 and 2662) were found to harbor CHG-DMCs and CHH-DMCs, respectively, and the majority of them harbored fewer than three CHG-DMCs and/or CHH-DMCs (Table S7B). Since the promoter, first exon, and first intron regions are of particular interest due to their specific DNA methylation status, we further found 5699 genes harboring ≥10 CpG-DMCs (CpG-DMC genes) and 210 genes harboring ≥3 CHG-DMCs and/or CHH–DMCs (CHG/CHH–DMC genes) at these regions (Table S7C). Functional annotation of the 5699 CpG-DMC genes indicated involvement in multiple biological processes, such as metabolic process, intracellular signal transduction, and cell migration, amongst others, as well as important immune-related pathways, such as leukocyte transendothelial migration, inflammatory mediator regulation, and bacterial invasion of epithelial cells. (Table S7D). However, the CHG/CHH–DMC genes were not significantly enriched in any GO term or KEGG pathway. This is consistent with the significance of DNA methylation at cytosine sites in the context of CpG and suggests their potential involvement in the regulation of host responses to SC subclinical mastitis.

### 2.2. Identification of Methylation Haplotype Blocks

**Figure 2:**
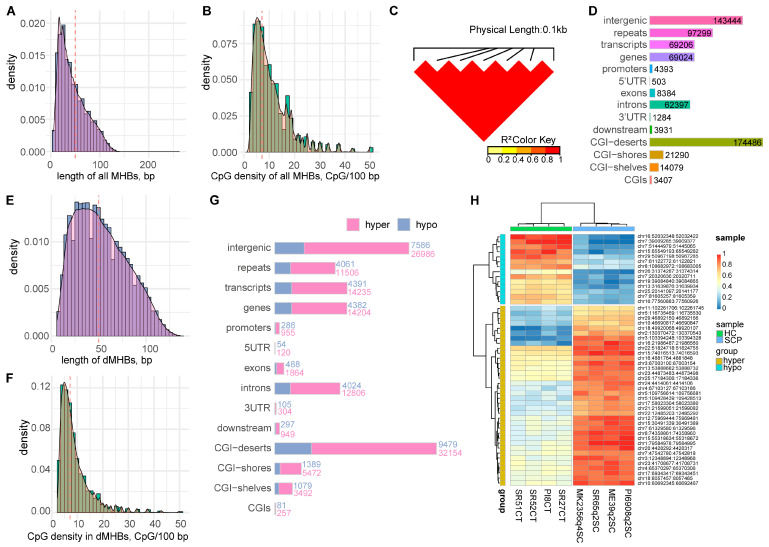
Characteristics of methylation haplotype blocks (MHBs). (**A**) Length distribution of all MHBs; (**B**) The distribution of CpG density in all MHBs; (**C**) Linkage disequilibrium (LD) score between CpG sites located in MHB chr1: 2,770,635–2,770,721 revealing the co-methylation status of adjacent CpG sites; (**D**) Count of MHBs that overlapped with genic regions and genomic regions; (**E**) Length distribution of differential MHBs (dMHBs); (**F**) The distribution of CpG density in dMHBs; (**G**) The dMHBs that overlapped with genic structures and genomic regions. The number at the right side of each bar indicates the number of hypo- (polo blue) and hyper- (tea rose) dMHBs that overlapped with the corresponding region; (**H**) A heat map of the top 50 most variable dMHBs showing clustering of SCP and HC groups. The red dotted line in (**A**,**B**,**E**,**F**) represents the median values of corresponding indicators displayed on the x-axis.

To better understand the DNA methylation pattern, we further identified a total of 212,299 methylation haplotype blocks (MHBs). MHBs capture the co-methylation between adjacent CpG sites. The MHBs were, on average, 45 bp long (ranging from 6 to 259 bp), with an average CpG density of 11 CpG/100 bp (Figure 2A,B). Most CpG sites inside the same MHBs were almost perfectly coupled (*r^2^*: ~1.0) (Figure 2C), suggesting their high co-methylation status. A large number of MHBs partly or completely overlapped with different genic structures and genomic functional regions (Figure 2D). For instance, 69,024 MHBs overlapped with genes and mostly within introns. A total of 4393 MHBs overlapped with promoters, including 128 MHBs harboring TSSs.

In order to quantitatively compare the methylation status of MHBs between SCP and HC groups, we first calculated the weighted methylation level or methylation haplotype load (MHL) for each MHB per sample. We then identified 53,098 differential MHBs (dMHBs) with significantly different MHL between the two groups (FDR < 0.05, |MHL difference| > 20%) (Table S8). The length of dMHBs ranged from 6 to 136 bp, with an average of 50 bp (Figure 2E). About 50% of dMHBs harbored three CpG sites, and the average CpG density of dMHBs was 7 CpG/100 bp (Figure 2F). Consistent with CpG-DMCs, about 75% of dMHBs showed higher methylation levels in the SCP group compared to the HC group (Table S8). The distribution of dMHBs was similar to CpG-DMCs (Figure 2G). For instance, the majority of dMHBs were found in intergenic regions and/or CGI deserts, and most dMHBs overlapping with genes were found in intron regions of genes. A total of 1241, 192, and 6188 dMHBs overlapped with 963 promoters, 163 first exons, and 2405 first introns, respectively. Moreover, the methylation status of the top variable dMHBs showed significant differences and a clear clustering of individuals according to their respective groups (SAP and HC) (Figure 2H), suggesting their possible association with SC subclinical mastitis. The functional enrichment of 3191 genes harboring dMHBs at their promoter, first exon, and/or first intron regions showed significant enrichment in biological processes related to cell migration and locomotion, and pathways related to immune functions, such as epithelial cell migration, epithelium migration, leukocyte transendothelial migration, and T cell receptor signaling pathway (Table S9). These biological processes and pathways are known to play important roles in innate and/or adaptive immunity during mammary gland infection and maintenance of mammary gland health, further highlighting the possible effects of DNA methylation changes during SC subclinical mastitis.

### 2.3. Global Methylome and Transcriptome Integration Indicates Negative Correlation

**Figure 3:**
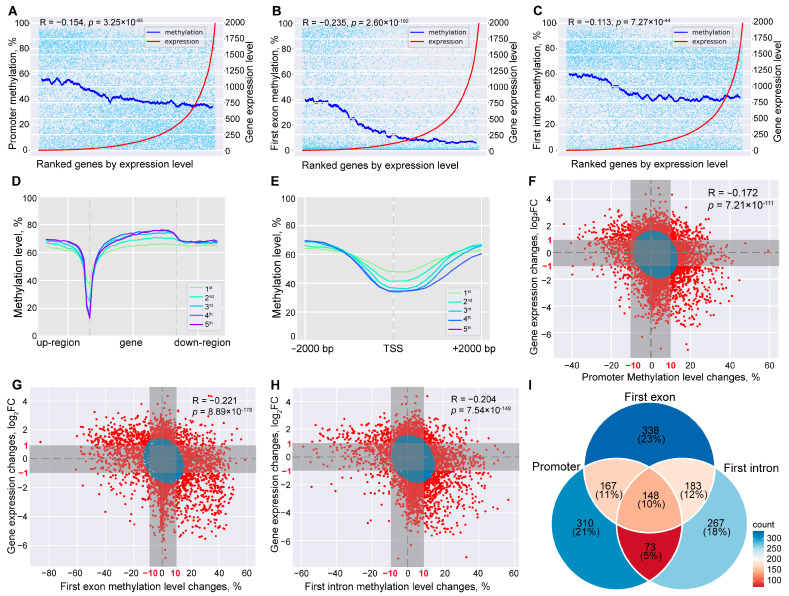
Integration of DNA methylation and mRNA transcriptome profiles. (**A**–**C**) Global negative correlation between gene expression level and the methylation level of promoter (**A**), first exon (**B**), and first intron (**C**) regions; (**D**) The methylation level trends around genes, where the up-region and down-region stand for the upstream and downstream region of genes, respectively, and their length is half the length of corresponding genes; (**E**) An expanded view of the methylation level trends of a 2000 bp region around transcription start sites (TSS); (**F**–**H**) Differentially methylated and expressed genes (DMEGs) with significant different methylation levels at promoter region (**F**), first exon (**G**), and first intron (**H**). (**I**) Venn plot showing the integration of DMEGs with significant methylation changes at promoter, first exon, and first intron.

For the purpose of investigating the association between DNA methylation and gene expression, we first calculated the methylation levels of genic structures (promoter, first exon, first intron, gene body, exons, and introns) for each gene as the arithmetic mean of the methylation level of all qualified CpG sites. The DNA methylation level of promoters, first exons, and first introns was significantly negatively correlated with gene expression levels at a scale of the whole genome (*p* < 5 × 10^−8^) (Figure 3A–C). Globally, the higher the expression level of genes, the lower the methylation level at their regulatory regions (promoter, first exon, and first intron). Furthermore, the classic valley-like changes in DNA methylation around TSSs also showed possible associations with the gene expression level, in that the higher the expression level of genes, the steeper their methylation level change is around TSSs (Figure 3D,E).

We next focused on the DNA methylation levels of the regulatory regions and identified significant negative associations between their DNA methylation changes and gene expression changes between SCP and HC (*p* < 5 × 10^−8^) (Figure 3F–H). Applying a Gaussian Mixture Model (GMM), we identified 2694 differential genes having significant changes in their gene expression level and/or promoter methylation level (*p* of GMM < 0.005) (Figure 3F). We further filtered the differential genes and only kept 698 genes having greater than 10% changes in promoter methylation level and 2 fold-changes in gene expression and referred to them as differentially methylated and expressed genes (DMEGs) (Figure 3F and Table S10A). Meanwhile, we used the same strategy and identified 836 and 671 DMEGs with significant changes in the methylation levels of their first exons and first introns, respectively (Figure 3G,H, Table S10B,C). By merging the promoter, first exon, and first intron DMEGs together, a total of 1486 DMEGs were obtained, including 148 DMEGs with significant changes in the methylation levels of all three regulatory regions during SC subclinical mastitis (Figure 3I and Table S10D). About 75% of the 1486 DMEGs showed opposite changes in their gene expression and DNA methylation levels (Table S10D). Among them, 761 DMEGs had down-regulated gene expression and hyper-methylation in their regulatory regions, while 374 DMEGs had up-regulated gene expression and hypo-methylated regulatory regions. In addition, we checked the distribution of dMHBs in the regulatory regions of DMEGs and found that 924 dMHBs overlapped with the regulatory regions of 322 DMEGs (Table S10D). Among them, 155 dMHBs overlapped with the promoter of 104 promoter DMEGs. Meanwhile, 52 and 444 dMHBs overlapped with the first exon and first intron of 40 first exon DMEGs and 133 first intron DMEGs, respectively (Table S10E). Notably, *PRSS8* and *VILL* are two genes with significantly differently methylated promoters, first exons, and first introns, which also overlapped with dMHBs in all of these regions (Table S10E).

### 2.4. DNA Methylation Alterations’ Implication in the Regulation of Host Responses to SC Subclinical Mastitis

**Figure 4:**
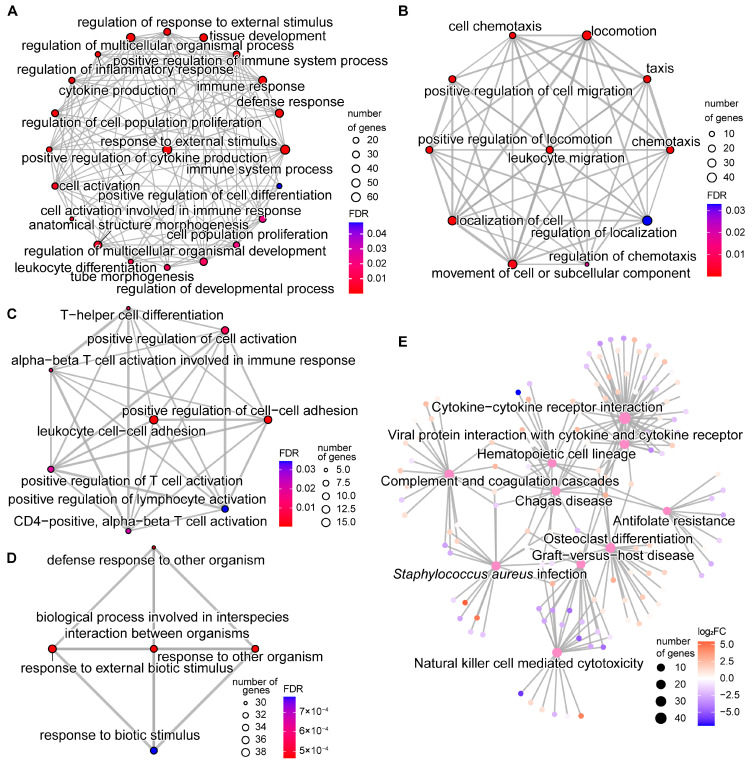
Functional enrichment for differentially methylated and expressed genes (DMEGs). (**A**–**D**) The biological processes gene ontology (BP-GO) terms significantly enriched by DMEGs, showing significant clustering based on similar functions. (**A**) The first cluster of BP-GO terms are processes related to immune responses and development processes; (**B**) The second cluster of BP-GO terms are processes related to cell activities, such as cell migration and movement; (**C**) The third cluster of BP-GO terms are relevant to T cell activities; (**D**) The firth cluster of BP-GO terms are processes related to defense responses to other organisms (the fourth cluster of BP-GO terms is found in Table S11A); (**E**) The KEGG pathways significantly enriched by DMEGs.

To explore the potential effects of DMEGs, we performed functional enrichment for the 1486 DMEGs, which demonstrated the significant enrichment of 86 GO terms, including 70 biological process (BP), 13 molecular function (MF), and 3 cellular component (CC) terms, as well as 10 KEGG pathways (Table S11A). The GO terms and KEGG pathways are mostly processes related to host responses to external stimuli and diseases, particularly immune functions. For instance, 34 out of 70 BP-GO terms are relevant to immune processes and responses to external stimulus processes (Table S11A). In addition, the 70 GO terms were hierarchically classified into 5 clusters based on their pairwise similarities, further revealing their possible roles in the host response to SC subclinical mastitis (Figure S1). The first cluster includes 21 BP-GO terms with crucial roles in the immune response to external stimulus and the regulation of cell activities related to tissue development (Figure 4A). For example, the top three most significantly enriched GO terms in this cluster are response to external stimulus (GO:0009605), immune system process (GO:0002376), and defense response (GO:0006952) (Table S11A). The second cluster of 11 BP-GO terms are processes related to chemotaxis, cell migration, and locomotion, such as leukocyte migration (GO:0050900), chemotaxis (GO:0006935), and positive regulation of cell migration (GO:0030335) (Figure 4B), which are important for recruiting immune and related cells from the blood into the mammary gland to fight infections. In addition, the third cluster are terms related to the positive activation of immune-related cells, especially T cells (Figure 4C), which have crucial immune functions, especially specific immunity. The fourth cluster includes BP-GO terms related to epithelial cell development, wound healing, and other system processes (Table S11A). The fifth cluster consists of five BP-GO terms related to responses to other organisms (Figure 4D). The five clusters suggest potential distinct regulatory roles for DMEGs in the mammary gland immune response to SC. Moreover, the ten enriched KEGG pathways further support the effects of DMEGs on mammary immune functions during SC subclinical mastitis (Figure 4E). For instance, over 50% of enriched pathways have immune-related functions, particularly the activities of cytokines, such as cytokine–cytokine receptor interaction (bta04060), viral protein interaction with cytokine and cytokine receptor (bta04061), and natural-killer-cell-mediated cytotoxicity (bta04650) (Table S11A). The other pathways are disease-related, including *Staphylococcus aureus* infection (bta05150), further implicating DMEGs in the pathogenesis of SC subclinical mastitis.

Moreover, we found a total of 122 DMEGs that were enriched in at least 3 BP-GO terms (Table S11B). Most of them were immune-related genes, such as *AIF1*, *BATF*, *CCL3*, *CXCL11*, *CXCL17*, *IL12B*, *IL1B*, *IL34*, *IL6*, *IRF6*, and *NLRP3* (Table S11B). Noteworthy is that about 78% of them (94) showed an inverse relationship in their gene expression and regulatory regions’ methylation level changes, including 43 DMEGs with a hypo-methylated regulatory region and up-regulated gene expression and 51 DMEGs with a hyper-methylated regulatory region and down-regulated gene expression. The majority of DMEGs enriched in immune functions BP-GO terms, particularly those of the third cluster associated with T cell activities and the fifth cluster associated with the defense response to other organisms, have up-regulated gene expression with hypo-methylation in their regulatory regions, such as *BATF*, *IL12B*, *IL6*, *HLX*, *AIF1*, *NLRP3*, *NOD2*, *FUT7*, and *CATHL3* (Table S11A,B). Most genes of the second cluster BP-GO terms associated with cell migration had down-regulated expression and hyper-methylation in their regulatory regions, such as *CXCL11*, *CXCL17*, *IL34*, *TMEM102*, *NFIB*, *FGFBP1*, *RAB13*, and *PGF*. Furthermore, 46 out of the 122 DMEGs harbored dMHB in their regulatory regions. For instance, the first intron of *B4GALT1*, involved in 14 immune-related BP-GO terms, including immune system process, defense response, and leukocyte migration, is collocated with 15 dMHBs (Table S11B).

**Figure 5:**
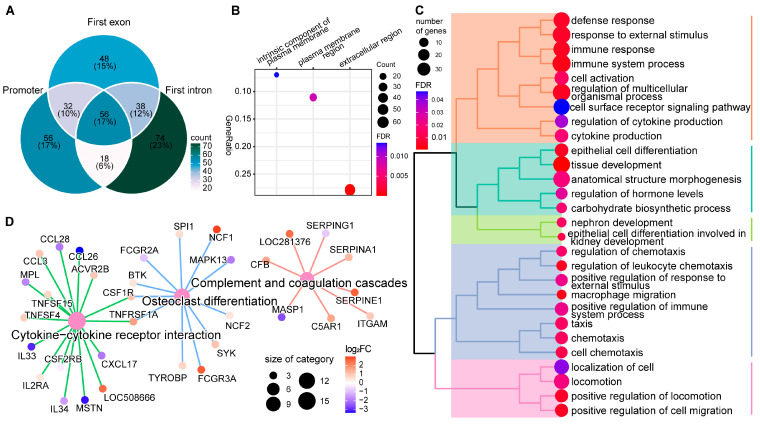
Functional enrichment for 322 differentially methylated and expressed genes (DMEGs) that overlapped with dMHBs. (**A**) Venn plot showing the integration of DMEGs which overlapped with dMHBs at their promoter, first exon, and first intron regions. (**B**) The three significantly enriched cellular component gene ontology (CC-GO) terms. (**C**) Tree plot of significantly enriched biological processes gene ontology (BP-GO) terms showing significant clustering based on pairwise similarities of the enriched terms. The orange color cluster (first from top) is related to immune and defense responses. The cyan (second from top) and green (third from top) clusters concern tissue development. The purple cluster (fourth from top) is related to chemotaxis processes and regulation of immune processes, while the pink cluster (fifth from top) is relevant to cell localization. (**D**) The significantly enriched KEGG pathways (large pink dots) and associated genes (smaller colorful dots).

Moreover, functional enrichment for 322 DMEGs having dMHBs at their regulatory regions (dMHB-DMEGs, Figure 5A) indicated significant GO terms (28 BP-GO and 3 CC-GO terms) and KEGG pathways (*n* = 3) (Figure 5B–D and Table S11C). The enriched terms for the 322 dMHB-DMEGs showed very high similarity with those of all 1486 DMEGs (about 75% of enriched GO terms were similar for the 2 DMEG sets, and all KEGG pathways enriched by the dMHB-DMEGs were also enriched by all DMEGs (Table S11C). For instance, three CC-GO terms enriched by dMHB-DMEGs (Figure 5B) were also enriched by all DMEGs. Furthermore, GO-terms macrophage migration (GO:1905517) and regulation of leukocyte chemotaxis (GO:0002688) were enriched by dMHB-DMEGs. Although all DMEGs were not found in these two BP-GO terms, they were among genes in related terms, such as leukocyte migration (GO:0050900), chemotaxis (GO:0006935), cell chemotaxis (GO:0060326), and leukocyte cell–cell adhesion (GO:0007159). Additionally, terms of the cluster of 28 BP-GO terms were similar to that of all DMEGs but more streamlined to show the involvement of the 322 dMHB-DMEGs with intense DNA methylation changes (dMHBs) in functional modules, including (1) the defense and immune response; (2) regulation of immune system process; (3) regulation of cell locomotion, particularly chemotaxis; and (4) tissue development (Figure 5C).

### 2.5. Identification of Candidate Discriminant Signatures Related to SC Subclinical Mastitis

**Figure 6:**
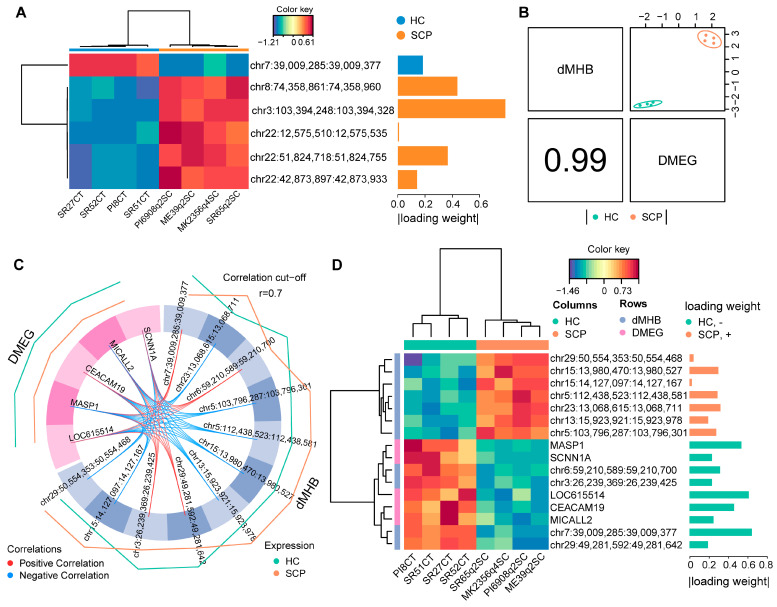
Identification of candidate discriminant signatures for SC subclinical mastitis. (**A**) Cluster and loading weight of six discriminant dMHBs selected by sPLS-DA. The orange and blue colors of the bar for absolute loading weight indicate higher methylation levels in SCP and HC groups, respectively. The greater the absolute loading weight, the more important the signature; (**B**) The strong correlation between the dMHBs and DMEGs discriminant signatures; (**C**) The significant and strong correlation (*r* > 0.7) between genetic and epigenetic discriminant signatures (11 dMHBs and 5 DMEGs) selected by DIABLO. The external lines indicate the methylation/expression level of corresponding signatures in SCP (cyan) and HC (orange) groups, while the outer line means a higher level; (**D**) Cluster and loading weight of discriminant signatures.

To identify relevant dMHBs driving the discrimination between SCP and HC groups, we applied the sparse PLS—Discriminant Analysis (sPLS-DA) [35] on 7560 dMHBs which overlapped with promoters, first exons, or first introns of genes. The analyses of sPLS-DA indicated six dMHBs as the candidate discriminant signatures that correctly classified the samples into SCP and HC groups (Table S12A). Five of the six dMHBs had higher methylation levels in the SCP group compared to the HC group, while dMHB chr7:39,009,285:39,009,377 (overlapped with the first intron of *DOK3*) was the only hypo-methylated signature (Figure 6A). The dMHB chr3:103,394,248:103,394,328 was the most important signature with the greatest absolute loading weight (0.7886), which showed a significantly higher methylation level in the SCP group than in the HC group. This dMHB harbored four CpG-DMCs and one DMR and overlapped with the first intron of *ZNF691*. The second and third important signatures were chr8:74,358,861:74,358,960 and chr22:51,824,718:51,824,755, which overlapped with the first intron of *STMN4* and *MAP4*, respectively. The dMHB chr22:51,824,718:51,824,755, 38 bp long, has the highest DMC density (nine CpG-DMCs) among the six dMHBs. The candidate signatures, dMHB chr22:42,873,897:42,873,933 and chr22:12,575,510:12,575,535, are both located on chromosome 22 and overlapped with the promoter of *KCTD6* and the first intron of *CX3CR1*, respectively.

In addition, we also introduced the core DIABLO method [35] to integrate the DNA methylation and gene expression datasets to identify the correlated epigenetic and genetic signatures that explain the difference between SCP and HC groups. We used the 924 dMHBs that overlapped with the regulatory regions of DMEGs and the 322 dMHB-DMEGs as input datasets for DIABLO, which showed a significantly strong correlation (*r* = 0.99) (Figure 6B). The results identified 11 dMHBs and 5 genes as the signatures driving the discrimination between SCP and HC groups (Table S12B). The signatures showed a strong correlation with each other (Figure 6C). It is worth noting that the most important epigenetic signature, dMHB chr7:39,009,285:39,009,377 (|loading weight| = 0.644), was also selected by sPLS-DA above, which is nearly perfectly methylated in the HC group but barely methylated in SCP group. Another three dMHBs signatures were also hypo-methylated in SCP group (~10% methylation in SCP and ~70% methylation in HC), including chr6:59,210,589:59,210,700 (overlapped with the first intron of *RHOH*), chr3:26,239,369:26,239,425 (overlapped with the promoter of *CD101*), and chr29:49,281,592:49,281,642 (overlapped with the first intron of *TSPAN32*). The remaining seven dMHBs signatures were all hyper-methylated (higher methylation levels in the SCP group) (Figure 6D). Interestingly, two hyper-methylated dMHBs (chr15:13,980,470:13,980,527 and chr15:14,127,097:14,127,167) both overlapped with the first intron of *MAML2* and harbored four and seven CpG-DMCs, respectively. Furthermore, hyper-methylated dMHBs also overlapped with the first introns of genes, including chr23:13,068,615:13,068,711 (*KCNK5*), chr5:112,438,523:112,438,581 (*ZC3H7B*), and chr13:15,923,921:15,923,978 (*GATA3*). Moreover, the hyper-methylated dMHBs, chr5:103,796,287:103,796,301 and chr29:50,554,353:50,554,468, overlapped with the 5′UTR of *LPAR5* and the promoter of *PNPLA2,* respectively. Furthermore, the five genetic signatures (*CEACAM19*, *LOC615514*, *MASP1*, *MICALL2,* and *SCNN1A*) all showed lower expression levels in the SCP group compared to the HC group as well as a strong positive correlation with the seven hyper-methylated dMHBs and negative correlation with the four hypo-methylated dMHBs (Figure 6C).

### 2.6. Two dMHBs Show Significant Associations with Mammary Gland Health and Milk Production in a Larger Sample Size

**Figure 7:**
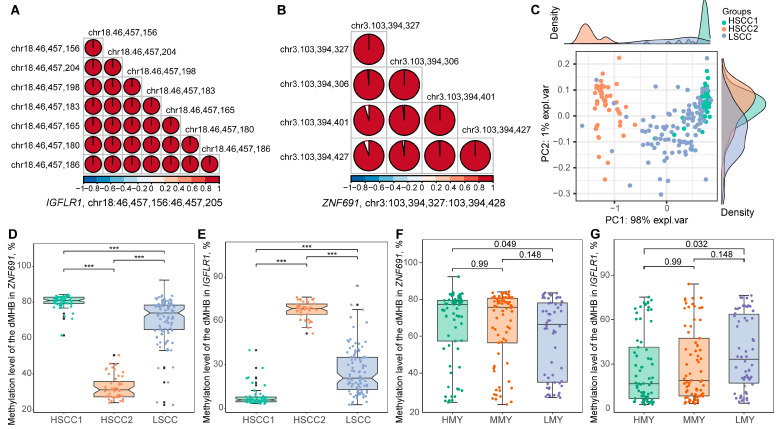
Validation of two dMHBs in a larger sample size (*n* = 200 cows). (**A**,**B**) The significant and strong correlation between CpG sites located in dMHBs chr18:46,457,156:46,457,205 (**A**) and chr3:103,394,327:103,394,428 (**B**), which overlapped with *IGFLR1* (promoter) and *ZNF691* (first intron), respectively; (**C**) PCA plot of methylation level of CpG sites detected in the two dMHBs showing clear clustering of cows, including clustering of HSCC cows into two groups- HSCC1 and HSCC2; (**D**,**E**) The significant different methylation levels of chr3:103,394,327:103,394,428 (**D**) and chr18:46,457,156:46,457,205 (**E**) between cows with different SCCs (proxy for mammary gland health condition). The high and low milk somatic cell counts are proxies for intramammary inflammation (HSCC1 and HSCC2) and good health (LSCC), respectively. “***” represents significant differences (*p* < 0.05) between corresponding groups; (**F**,**G**) The difference in the methylation level of chr3:103,394,327:103,394,428 (**F**) and chr18:46,457,156:46,457,205 (**G**) between cows with different milk yields, including high (HMY, ≥40 kg/day), median (MMY, 30–40 kg/day), and low (LMY, ≤30 kg/day) milk yields.

To further validate the observed signatures of dMHBs, we selected two dMHBs and tested their performance in a larger sample size. We sampled 200 cows, including 100 with high SCC (HSCC) and 100 with low SCC (LSCC), from 9 commercial dairy farms to further investigate the DNA methylation changes in 2 dMHBs (Table S13A). The DNA methylation changes of the selected dMHBs were quantified via target bisulfite amplicon sequencing (TBAS), followed by an investigation of their association with mammary gland health (SCC and linear score of SCC (SCS)) and milk production (test day milk yield (MY), milk protein (MP), and milk fat (MF) percentage) traits (Table S13B). The first dMHBs was the most important candidate signature, chr3:103,394,248:103,394,328, selected by sPLS-DA. This dMHB (chr3:103,394,327:103,394,428) was merged with a closely located dMHB to form one region (chr3:103,394,248:103,394,428) for the TBAS. A total of 4 CpG sites were successfully sequenced for this region in the 200-cow sample. The second dMHB chr18:46,457,156:46,457,205 (hypo-methylated, harbored seven CpG-DMCs, and overlapped with the promoter of *IGFLR1* and exon of *U2AF1L4*) was randomly selected. Consistent with the identification of dMHB, the CpG sites detected inside the same dMHB in 200 cows showed significant strong correlation between each other (*r* > 0.8) (Figure 7A,B), thus substantiating the fact that MHB/dMHB measured the co-methylation between adjacent CpG sites well. Interestingly, the PCA plot shows a clustering of the HSCC cows into two groups based on the methylation status of the two dMHBs (eleven CpG sites), which had significantly different methylation levels (Figure 7C and Table S13C). Therefore, we separated the HSCC group into two subgroups, HSCC1 (*n* = 52) and HSCC2 (*n* = 48), for subsequent statistical comparisons.

First, all eleven CpG sites showed significantly different methylation levels between HSCC1/HSCC2 and LSCC groups (Table S13D). We next calculated the arithmetic mean of detected CpG sites to represent the methylation level of the two dMHBs. Consistently, dMHB chr3:103,394,327:103,394,428 and chr18:46,457,156:46,457,205 were significantly differentially methylated among the three groups (Figure 7D,E, Table S13E). It is noteworthy that the methylation changes of these two dMHBs in the HSCC1 group compared to the LSCC group, including the higher methylation level of chr3:103,394,327:103,394,428 (Figure 7D) and lower level of chr18:46,457,156:46,457,205 (Figure 7E), were similar with that between the SCP and HC group detected by WGMS data. However, the methylation levels of these two dMHBs in HSCC2 are extremely different from the HSCC1 group, and their changes compared with LSCC are opposite to the HSCC1 group, which also means that they are opposite to WGMS results. Accordingly, we speculate that cows in the HSCC1 group may have similar mammary gland conditions to those in the SCP group, such as subclinical mastitis. Meanwhile, the high SCC (mean = 734,291 cells/mL) in cows of the HSCC2 group may be caused by other factors and deserves further exploration. We next tested the association between the two dMHBs and mammary gland health indicators, revealing their significant associations with SCC and SCS (Table S13E). For cows in the HSCC1 and LSCC group, chr3:103,394,327:103,394,428 and chr18:46,457,156:46,457,205 had negative and positive associations with SCC/SCS, respectively (|r| > 0.2 and *p* < 0.05), and their association with SCS was stronger. On the contrary, cows of HSCC2 and LSCC group showed SCC/SCS association with these two dMHBs in the opposite direction compared to HSCC1, including positive association with chr3:103,394,327:103,394,428 and negative association with chr18:46,457,156:46,457,205 (Table S13E).

Moreover, we regrouped cows according to their milk traits, including milk yield (MY), milk protein, and fat percentage, to investigate the association between the two dMHBs and milk production performance. The methylation levels of the two dMHBs showed no significant differences between high and medium MY (HMY and MMY) as well as between medium MY and low MY (MMY and LMY) (Figure 7F,G). However, the methylation levels between HMY and LMY were significantly different (FDR < 0.05) (Figure 7F,G). As shown in Figure 7F, the methylation level of dMHB chr3:103,394,327:103,394,428 in HMY was significantly higher than in the LMY group (FDR < 0.05). Meanwhile, dMHB chr18:46,457,156:46,457,205 showed a significantly lower methylation level in the HMY group than in the LMY group (FDR < 0.05) (Figure 7G). This suggests that the dMHBs related to subclinical mastitis may paly roles in the regulation of mammary gland performance for milk production. However, we did not find significant differences in the methylation levels of these two dMHBs among cows with different milk fat or protein percentages (Table S13F).

## 3. Discussion

In this study, we report abundant DNA methylation changes at the scale of the whole genome and integration with the mRNA transcriptome of milk somatic cells during bovine SC subclinical mastitis. To the best of our knowledge, this is the first study to investigate the global DNA methylation patterns and changes related to bovine subclinical mastitis caused by a predominant CNS such as SC. Abundant DNA methylation changes related to SC subclinical mastitis were identified. Therefore, we believe our study supports the hypothesis that DNA methylation may be an important regulatory mechanism underlying the host response to SC subclinical mastitis and provide good resources for further epigenetic investigations.

In this study, we built a compressive landscape of DNA methylation changes related to SC subclinical mastitis. In addition to the classical methods of identifying DNA methylation changes such as DMCs and DMRs, we also introduced the method of MHBs, which measured not only the methylation level of individual cytosines but also the co-methylation between adjacent CpG sites [35]. Using the principle behind MHB, it is possible to avoid the potential bias caused by the technical noise of measuring methylation levels of single cytosines or the subjective choice of window and length specification for DMR. The average size of dMHBs identified in this study was 53 bp (ranging from 6 to 136 bp), which is much smaller than the size of 1000 bp commonly set for DMR analysis [36]. Furthermore, the average MHB size in this study (median of 39 bp; mean of 45 bp) is similar to the size of MHBs identified in bovine sperm (average of 52 bp) [37] but shorter than the size of MHBs identified in mixed human tissues (average of 95 bp) [38]. A shorter dMHB length may have the advantage of being more specific since short regions are more likely to only overlap or associate with individual genes or regulatory elements, and consequently could help in the identification of the specific biological processes or pathways they really affect. The shorter length of dMHBs also has some technical advantages as they could be more easily and accurately measured using certain sequencing or assay technologies such as TBAS and liquid hybridization capture-based bisulfite sequencing approaches, which support the study of target regions up to 200 bp [39,40], which is more practical in population analyses.

Although the possible roles of DNA methylation have not been well studied in bovine mastitis due to CNS, particularly SC, DNA methylation changes have already been identified as one potential regulatory mechanism of mastitis caused by other major pathogens, such as *S. aureus*, *S. uberis*, and *Escherichia coli* (*E. coli*) [41,42,43,44]. Higher DNA methylation level has been found in cows with *S. aureus* subclinical mastitis [45,46], which is consistent with the global higher methylation level detected in the SCP group in this study. On the contrary, lower global methylation levels have been reported in dairy cows with *S. uberis* subclinical mastitis [41] and *E. coli* mastitis [44]. It is not surprising that DNA methylation changes related to SC subclinical mastitis showed similarity with *S. aureus* subclinical mastitis because they both belong to *staphylococci*, the most commonly isolated pathogens from subclinical mastitis cases in most countries [3,47]. Moreover, they are both capable of forming biofilms in the mammary gland tissue, making them hard to eliminate and thereby causing recurrent infections [48]. In addition, about 72% of DMRs (42,263), 38% of dMHBs (20,415), 61% of CpG-DMCs, and 11% of CHG/CHH-DMCs in this study are common (same genomic locations and lengths) with those found in *S. aureus* subclinical mastitis cows [46]. Interestingly, about 99% of the common DMCs, DMRs and dMHBs showed methylation changes in the same directions (hyper- or hypo-methylated) between the two studies, further suggesting/supporting similarities in the mode of infection by the pathogens, DNA methylation changes, and host response to SC and *S. aureus* infections.

Integration analyses between DNA methylation and transcriptome profiles revealed a global negative correlation between DNA methylation at the promoter and gene expression, which is consistent with the well-known repression effects of DNA methylation at the promoter on transcription activities [49,50,51]. We also identified negative correlations between gene expression and methylation of the first exon and first intron, which is not often reported but may affect gene expression by other mechanisms, such as alternative transcriptional silencing [52,53,54]. Moreover, DNA methylation has been reported to be a transcriptional regulator of the immune system, with important roles during disease progression [21,55]. In our study, the methylation changes in these regulatory regions were found to be negatively correlated with the gene expression changes during SC subclinical mastitis at the scope of the whole genome, supporting our earlier observations in response to subclinical mastitis due to *S. aureus* [46]. At individual gene levels, the majority of DMEGs had inverse changes in their gene expression and DNA methylation, further suggesting the potential of DNA methylation changes as a regulatory mechanism underlying the transcriptional activities of genes during SC subclinical mastitis. Interestingly, these DMEGs were significantly enriched in many biological processes and pathways related to immune functions, indicating their possible effects on the mammary gland defense and immune responses during SC subclinical mastitis.

Consistently, the functional enrichment suggested the possible involvement of DNA methylation changes in the regulation of host responses to SC subclinical mastitis. For example, enriched KEGG pathways via DMEGs were closely related to immune functions and diseases, such as cytokine–cytokine receptor interaction, natural-killer-cell-mediated cytotoxicity, and *Staphylococcus aureus* infection pathways. Furthermore, significantly enriched BP-GO terms suggest that DMEGs are involved in many biological processes which are crucial for the host response to SC subclinical mastitis. Multiple biological processes of the immune system process, immune response, and defense response to other organisms, which would be triggered in response to the invasion of SC into the mammary gland, were enriched by DMEGs. It is worth mentioning the cluster of biological processes terms related to the positive regulation of T cell activities, which are involved in recognizing bacterial antigens and initiating the immune response, particularly adaptive immunity [56]. During mastitis, T cells play important roles in coordinating and regulating the activity of other immune-related cells, such as B cells, macrophages, and neutrophils, which are critical for controlling and resolving the infection in the mammary gland [57,58]. Interestingly, the expression of most DMEGs involved in these processes was up-regulated with hypo-methylation at their regulatory regions, suggesting that the decreased DNA methylation may mediate the up-regulated expression of related genes and thereby promote the immune defense against SC invasion. This is consistent with a previous finding that DNA methylation contributes to enhancing the expression of immune-related genes in response to mastitis [44]. On the other hand, DMEGs’ involvement in biological processes related to cell migration and locomotion suggests their participation in the recruitment of immune-related cells from the bloodstream into the mammary gland (the site of infection). Furthermore, most DMEGs involved in biological processes terms such as epithelial cell development and other system process were down-regulated, and their regulatory regions were hyper-methylated. The down-regulated expression of these genes may contribute to the disruption of the epithelial barrier in the mammary gland and making it more vulnerable to bacterial infections, which may explain the long course of SC subclinical mastitis. Similarly, the enhanced expression of immune-related genes and decreased expression of genes related to tissue development have also been identified in milk somatic cells in response to *S. aureus* subclinical mastitis [59]. In summary, the DNA methylation changes are presumably involved in mediating transcription activities of important genes and, thereby, in the regulation of the host responses to SC subclinical mastitis.

Using the sPLS-DA and DIABLO methods, we further identified 16 dMHBs as candidate discriminant signatures of SC subclinical mastitis. For instance, the hypo-methylated dMHB chr7:39,009,285:39,009,377, which overlapped with the first intron of *DOX3*, was identified by both sPLS-DA and DIABLO methods as the candidate signature of SC subclinical mastitis. *DOK3* was found to act as a negative regulator of several signaling pathways, including those activated by T cell receptors and cytokine receptors, such as B cell receptor signaling pathway [60]. Plus, the significant DNA methylation changes at its promoter were also identified in relation to *S. uberis* subclinical mastitis [41], suggesting its possible involvement in the regulation of subclinical mastitis. The other dMHB signatures also overlapped with genes, whose expressions are found to be important for the health of dairy cows. For instance, the differential expression of genes (*CX3CR1*, *RHOH*, *LPAR5*, and *GATA3*) overlapped with dMHB discriminant signatures related to SC subclinical mastitis have been found relevant to immune responses to bovine mastitis [61,62,63,64,65]. Moreover, *KCNK5*, whose abnormal expression level has been associated with mastitis and heat stress in mouse/rat modules [66,67], harbored a dMHB signature in its first intron. For many of the genes that overlapped with dMHB discriminant signatures in this study, such as *ZNF691*, *MAML2*, *ZC2H7B*, *CD101*, *TSPAN32*, and *MAML2*, this is the first report of their involvement in bovine mastitis. Interestingly, our further investigation of two dMHBs signatures, including the one in the first intron of *ZNF691* in a larger population (200 cows), revealed significant strong associations with mammary gland health (SCC and SCS), suggesting their potential involvement in bovine mammary gland health. This observation further supports the exploration of the roles of identified discriminant dMHBs signatures and the evaluation of their potential as markers of SC subclinical mastitis.

While this study provides valuable insights into the possible contribution of DNA methylation to the regulation of SC subclinical mastitis, it is important to acknowledge its limitations. The first limitation concerns the composition of milk somatic cells analyzed in this study, which is composed of different cell types and which changes in response to infection. Knowing that DNA methylation has high tissue specificity, the method (whole-genome methylation sequencing, WGMS) used in its characterization in this study does not allow associating the DNA methylation alterations with specific cell types. This may have affected the reliability of results in this study and deserves further investigation using more advanced technologies, such as single-cell sequencing. Moreover, while this study provides important information about DNA methylation patterns and significant changes in association with SC subclinical mastitis, it is important to note that the sample size (four samples per group) was relatively small, and thus a larger sample size is needed to validate our findings. Importantly, our study detected DNA methylation alterations associated with SC subclinical mastitis, and the validation of two dMHBs in a larger sample size supports the stability of dMHBs related to mammary gland health and the reliability of our results. Since we did not perform a bacteriological examination on the samples used in the validation study, we could not further investigate the cause of the extreme methylation differences between the two subgroups of HSCC cows. Therefore, further validation of the dMHBs in samples with detailed information on health status and production traits is necessary to verify their potential as markers for SC subclinical mastitis. Finally, the DNA methylation changes found in this study contribute to a better understanding of the molecular mechanisms underlying SC subclinical mastitis and may ultimately lead to the development of improved strategies for the management of SC mastitis with significant economic benefits for the dairy industry.

## 4. Materials and Methods

### 4.1. Animals and Samples

Four commercial dairy farms in Quebec (Canada) with a history of intra-mammary infections were monitored for the identification of lactating Canadian Holstein cows with naturally occurring subclinical mastitis due to *Staphylococcus chromogenes* (SC). First, we monitored the health condition of 332 lactating cows by checking the milk somatic cell count (SCC) records from dairy herd improvement (DHI) records for a period of six months. The DHI records were obtained by analyzing monthly milk samples (20 mL composite sample/cow) for their contents of SCC and milk yield and composition (milk fat and protein percentages) by Lactanet (Lactanet Laboratories, Ste-Anne-de-Bellevue, QC, Canada). We found 74 cows that had high SCC (>350,000 cells/mL) for ≥ three consecutive months but without any clinical mastitis symptoms indicative of subclinical infection. Meanwhile, we identified 56 cows with low SCC (<150,000 cells/mL) for ≥ three months consecutively. An approximately 5 mL milk sample from each quarter of cows with high SCC and a composite sample (equal volumes from each quarter) from cows with low SCC were aseptically collected and sent to Biovet (Biovet laboratories, St-Hyacinthe, QC, Canada) for bacteriological examination on the same day of collection. A total of 7 cows with high SCC were identified to be positive only for SC (SCP) in one or more quarters, and 16 cows with low SCC were negative for all mastitis pathogens tested. We selected 5 cows from the 16 cows that were negative for pathogens for final sampling by keeping those with similar backgrounds (farm, parity, and lactation stage) with SCP cows. Then, we collected about 200 mL of milk samples from one positive quarter of SCP cows and 200 mL of composite milk (100 mL/quarter) from each negative cow. In cases where the mammary gland health condition changed between the initial bacteriological examination and actual sampling (about two to three days), 2 mL of each final milk sample was again sent for bacteriological examination. Finally, samples from four SCP cows (SCP group) and four healthy cows (HC group) with consistent results were retained for the next-steps analyses (Table S14).

### 4.2. DNA and RNA Isolation from Milk Somatic Cells

Final milk samples were placed on ice after collection and immediately transported back to our laboratory for isolation of milk somatic cells via low-speed centrifugation (1500× *g* for 15 min at 4 °C). After removing the fat layer at the top, we decanted the whey layer, leaving about 2 mL of the precipitate containing milk somatic cells at the bottom of the tube. The precipitate with cells was washed twice by the addition of 40 mL phosphate-buffered saline (PBS) and centrifugation (1500× *g* for 15 min at 4 °C). The washed milk somatic cells were resuspended in 1 mL PBS and split into two parts. One part was stored at −20 °C for DNA isolation, while Trizol reagent (~1 mL) was added to the other part and stored at −80 °C for RNA isolation.

DNeasy Blood and Tissue Kit (Qiagen Inc., Toronto, ON, Canada) was used to isolate genomic DNA from washed cells and quantified using Quant-iT™ PicoGreen^®^ dsDNA Assay Kit (Life Technologies, Burlington, ON, Canada). RNeasy Mini Kit (Qiagen Inc., Toronto, ON, Canada) was used to isolate total RNA according to the manufacturer’s instructions. Then, the quantity and integrity of isolated total RNA were checked using Agilent Bioanalyzed 2100 (Agilent Technologies, Saint-Laurent, QC, Canada) and LabChip GXII (PerkinElmer Inc., Waltham, MA, USA) instruments, respectively. RNA samples with an RNA integrity number >7 were further processed.

### 4.3. Whole Genome-Wide DNA Methylation Sequencing and Data Processing

Genomic DNA was used to prepare whole genome methylation sequencing (WGMS) libraries using NEBNext^®^ Enzymatic Methyl-seq kit (New England BioLabs Ltd., Whitby, ON, Canada), which is an enzymatic-based approach with higher accuracy and reliability than whole-genome bisulfite sequencing [68]. Then, libraries were quantified using Kapa Illumina GA with Revised Primers-SYBR Fast Universal kit (Kapa Biosystems Inc., Wilmington, MA, USA). The normalized libraries were equimolar pooled together and denatured in 0.05 mol/L NaOH and neutralized using HT1 buffer. Each pool was loaded at 225 pM on an Illumina NovaSeq S4 lane using Xp protocol with high sequencing depth (~40×). The run was performed at paired-end mode for 2 × 100 cycles. Library construction and sequencing were performed by Centre d’expertise et de services Génome Québec (https://cesgq.com/, accessed on 30 October 2021).

We used the standard pipeline for DNA methylation sequence data from nf-core (https://nf-co.re/methylseq (accessed on 30 October 2021), version 1.6.1) to process the raw WGMS data. Briefly, we selected the ‘EM Seq’ trimming profile to avoid the possible bias near the ends of reads (8 bp) caused by DNA end repair during library construction. FastQC (version 0.11.9) and Trim Galore! (Version 0.6.6) were used for sequence quality reports and for removing low-quality reads and adapter sequences, respectively. Then, the clean reads with high quality were merged and mapped to the bovine reference genome (ARS-UCD1.2, https://www.ncbi.nlm.nih.gov/genome/?term=ARS-UCD1.2 (accessed on 30 October 2021)) using Bismark (version 0.22.0). The function ‘bismark_methylation_extractor’ from Bismark was used to identify methylation and methylation sites in the context of CpG, CHG, or CHH (H stands for A, C, or T), which were identified in all samples and sites with ≥7× coverage depth per sample were retained for the next-step analysis.

To identify differential methylation sites, we used Methylkit (version 3.12) to compare the methylation levels of each cytosine in the context of CpG, CHG, and CHH between SCP and HC groups. Before the comparison, the background information of cows, including parity, lactation stage, and farms, were included as batch factors to model their potential effects to decrease random noise. The differentially methylated cytosine (DMC) was defined using two thresholds: adjusted *p* value (*q* value) < 0.05 and methylation level difference between groups ≥20%. Moreover, we used 1 kb wide window to scan the whole genome with a 1 kb step for identifying differentially methylated regions (DMRs). Three thresholds were used to select DMRs, including (1) *q* value < 0.05, (2) difference in average methylation level of CpG sites inside corresponding region between two groups ≥20%, and (3) harboring at least three CpG-DMCs.

### 4.4. Identification and Comparison of Methylation Haplotype Blocks

To better understand the methylation landscape, we used MONOD2 to identify methylation haplotype blocks (MHBs) in the bovine genome [38]. First, we pooled the clean WGMS reads of all samples together and used them to split the bovine reference genome (ARS-UCD1.2) into sequencable, mappable, and non-overlapping segments. Then, we identified the methylation haplotypes based on mapped reads within each segment. Next, the MHBs were identified by calculating the methylation linkage disequilibrium between combined methylation haplotypes. We filtered the MHBs by only keeping those that harbored ≥3 CpG sites and in which the r^2^ value of any two adjacent CpG sites was ≥0.5.

To compare the methylation status of MHBs between SCP and HC groups, we calculated the methylated haplotype load (MHL) for each MHB per sample. The MHL refers to the normalized methylation level of methylation haplotypes at different lengths. Then, we used a two-tailed Student’s *t*-test to compare the MHL of each MHB between the two groups. The *p* value was adjusted by Benjamini and Hochberg’s false-discovery rate (FDR) [69]. The significant differential MHB (dMHB) between SCP and HC groups was filtered by FDR < 0.05 and the difference in MHL between the two groups ≥20%.

### 4.5. RNA Sequencing and Raw Data Processing

To prepare the RNA sequencing libraries, 125 ng of total RNA per sample was subjected to ribosomal RNA depletion with QIAseq FastSelect Kit (Qiagen Inc.). The cDNA was then generated using NEBNext RNA First Strand Synthesis and NEBNext Ultra Directional RNA Second Strand Synthesis Modules (New England BioLabs). We proceeded with the next steps of library preparation according to the guidelines of NEBNext Ultra II DNA Library Prep Kit for Illumina (New England BioLabs). Next, we used Kapa Illumina GA with Revised Primers-SYBR Fast Universal kit (Kapa Biosystems) and a LabChip GXII (PerkinElmer) instrument to check the quantity and average fragment size of libraries. We then normalized and pooled libraries of all samples in equimolar proportions and loaded them at 200 pM on an Illumina NovaSeq S4 lane using Xp protocol. The sequencing was run for 2 × 100 cycles in a paired-end mode. Library construction and sequencing were performed by Centre d’expertise et de services Génome Québec (https://cesgq.com/).

After sequencing, base calling was performed using the RTA v3.4.4. program, and bcl2fastq2 v2.20 was used to demultiplex samples and generate reads. The standard bioinformatics pipeline for RNA sequencing data from nf-core (https://nf-co.re/rnaseq (accessed on 5 December 2021), version 3.3) was used to process raw sequencing data. Briefly, Trim Galore! (Version 0.6.5) was used to remove adapter sequences and reads with low quality. The clean reads were then aligned to the bovine reference genome ARS-UCD1.2 using STAR (version 2.7.1a). Salmon was used for downstream quantification with BAM-level and UMI-tools for deduplication. DESeq2 (version 1.34.0) was used to normalize read counts and identify genes by modeling the possible batch effects of parity, lactation stage, and farm.

### 4.6. Methylome and Transcriptome Data Integration

The MethGET program was used for the integration of methylome and transcriptome data to explore the possible relationship between DNA methylation and gene expression [70]. First, three input files, including the CGmap file of qualified CpG sites (methylome), normalized gene expression file (transcriptome), and the RefSeq assembly accession (GCF_002263795.1) of the reference genome, were assembled. Second, we calculated the general methylation level of the different gene structures, including promoter, first exon, first intron, exons, introns, and gene body for each gene, as the average methylation level of all CpG sites in each of the genic structures. Then, we checked the global association between DNA methylation of different genic structures and gene expression level at a scope of the whole genome by ‘Single-methylome analyses’, which were completed per sample. Next, we performed group comparison to check the association between DNA methylation changes in different genic structures and expression changes of corresponding genes at the genome-wide scale using Person’s correlation analysis. Finally, we checked the methylation status and gene expression levels of all genes identified using the Gaussian Mixture Model (GMM) from the scikit-learn package. Then, we identified genes with significant changes in the methylation level of their regulatory genic structures (promoter, first exon, or first intron) and their expression levels (CMM *p*-value < 0.005, |log2FC| ≥ 1 and methylation level difference ≥10%), which we referred to as differentially methylated and expressed genes (DMEGs) in this study. It is worth noting that GMM was performed to identify DMEGs based on the methylation levels of different regulatory genic structures and the expression level of corresponding genes separately, which yielded three lists of DMEGs with significant changes in the methylation level of the promoter (promoter DMEGs), first exon (first exon DMEGs), and first intron (first intron DMEGs), respectively.

### 4.7. Identification of Important Signatures for SC Subclinical Mastitis

First, we introduced the sparse PLS-DA (sPLS-DA) method from R package MixOmics (version 6.22.0) for dMHBs at regulatory regions, including promoters, first exons, and first introns to select a small subset of dMHBs relevant to characterize and discriminate SCP and HC groups. The methylation level (MHL) of selected dMHBs and the health condition (SCP or HC) were used as the inputs. We chose two key parameters (number of components and number of signatures to select) according to the instructions of the package [35]. First, we set the number of components as one because we had two groups (SCP and HC), and K-1 components (K: number of groups) was suggested to obtain the best classification performance. Then, we performed repeated cross-validation (4-fold cross-validation, 50 times repeat, and maximum prediction distance) using the ‘tune.splsda’ function to determine the suitable number of signatures.

In addition, DIABLO (core method from MixOmics), based on the improved implementation of the multivariate methodology Generalised Canonical Correlation Analysis (GCCA) and used to integrate different ‘OMICs’ data measured on the same biological samples [35], was used to integrate the methylome and mRNA transcriptome data and identify few highly correlated candidate signatures that explain the categorical difference between SCP and HC groups. The methylation status or methylation haplotype load (MHL) of dMHBs that collocated in the regulatory regions of DMEGs and the expression levels of corresponding DMEGs were used as the input for DIABLO to represent two ‘OMICs’ sources (DNA methylation alteration and gene expression changes), respectively. We chose a fully weighted design to balance the maximizing correlation between input datasets and the discrimination of signatures. We chose the two key parameters (number of components and number of signatures to select) that are similar for both methods (sPLS-DA and DIABLO). Instead of ‘tune.splsda’, the ‘tune’ function was used for the repeated cross-validation to determine the number of signatures to choose from each dataset.

### 4.8. Functional Analyses

We downloaded the genome structure annotation files of the bovine reference genome from the UCSC Table browser (https://genome.ucsc.edu/cgi-bin/hgGateway (accessed on 30 October 2021)), including the information about genes, repeat elements, and CpG islands (CGIs). The promoter region was defined as the region 2000 bp upstream of the TSS. Meanwhile, the downstream region was defined as the 2000 bp region downstream of the transcription termination site (TTS). We defined the left and right CGI shores as the 2000 bp region upstream and downstream of CGIs, respectively. Accordingly, the left (right) CGI shelf was the 2000 to 4000 bp region upstream and downstream of the corresponding CGI. The information about repeat elements was downloaded from the track ‘RepeatMasker’. We used the R package, annotatr (version 3.12), to check the collocation of DMCs, DMRs, MHBs, and dMHBs with the genic structures and genomic regions described above.

We performed functional enrichment analysis for specific gene sets, such as genes harboring DNA methylation changes (DMCs/dMHBs) and DMEGs using the R package ClusterProfiler (Version 4.2.2). Due to the parent–child structure of GO terms, we applied a ‘simplified’ strategy to reduce the redundancy of enriched GO terms. We only kept GO terms or KEGG pathways with adjusted *p* value (FDR) < 0.05 as the significantly enriched terms.

### 4.9. Validation of Candidate Discriminant dMHB Signatures

To validate the DNA methylation alterations within dMHBs, 200 cows from 9 commercial dairy farms in Quebec, Canada, with a high SCC (HSCC, >200,000 cells/mL, *n* = 100) or low SCC (LSCC, <100,000 cell/mL, *n* = 100) according to their DHI records were sampled. A milk sample of about 200 mL was collected from each cow in the HSCC (indicative of possible intramammary infection (IMI)) and LSCC (indicative of a healthy mammary gland) groups (Table S13A). We used the same methods described above to process the somatic milk cells and isolate and qualify DNA. Two dMHBs (chr18: 46,457,151-46,457,210, found within the promoter of *IGFLR1*) and chr3:103,394,248:103,394,428 (found within the first intron of *ZNF691*) were selected for validation using the method of target bisulfite amplicon sequencing (TBAS) based on next-generation sequencing technology. Briefly, primers for the two dMHBs were designed using MethPrimer (Table S13B). A total of 1 μg of genomic DNA per sample was bisulfite converted using the ZYMO EZ DNA Methylation-Gold Kit (Zymo Research, Irvine, CA, USA). Next, one-twentieth of the elution product was used as a template for PCR amplification (35 cycles) using KAPA HiFi HotStart Uracil+ ReadyMix PCR Kit (Kapa Biosystems, Wilmington, MA, USA). The PCR products were then pooled in equimolar proportions per sample, followed by being 5′-phosphorylated, 3′-dA-tailed, and ligated to the barcoded adapter using T4 DNA ligase (New England Biolabs, Ipswich, MA, USA). Finally, the barcoded libraries were sequenced on an Illumina Hiseq platform (paired-end,150 bp).

The raw TBAS data were processed by filtering to remove the adapter and low-quality sequences with Trimmomatic (version 0.36). Next, clean reads were aligned to the bovine reference genome (ARS-UCD1.2) using BSMAP (version 2.73). Then, the beta value of each detected CpG site was calculated and compared between groups using a two-tailed Student T test. The *p* value was adjusted using Benjamini and Hochberg’s false-discovery rate (FDR) correction [69], and FDR < 0.05 was defined as a significant difference. We further checked the correlation between CpG sites inside each dMHBs using the R package EnMCB (version 1.10.0). Finally, each dMHB was correlated to mammary gland health (SCC) and milk production (milk yield, milk protein, and fat percentage) traits using the R package CpGassoc (Version 2.60).

## Data Availability

The data presented in this study are openly available at the NCBI Sequence Read Archive (SRA) under the BioProject PRJNA962099 (WGMS) and PRJNA967255 (RNA-Seq).

## Supplementary Materials

The supporting information can be downloaded at: https://www.mdpi.com/article/10.3390/ijms241210369/s1.
