# Supplementary figures and images for: Genome-Wide DNA Methylation and Transcriptome Integration Associates DNA Methylation Changes with Bovine Subclinical Mastitis Caused by Staphylococcus chromogenes
Source: Int J Mol Sci. 2023 Jun 20;24(12):10369. doi: 10.3390/ijms241210369 (PMC10299661; doi:10.3390/ijms241210369)

# Supplementary Materials

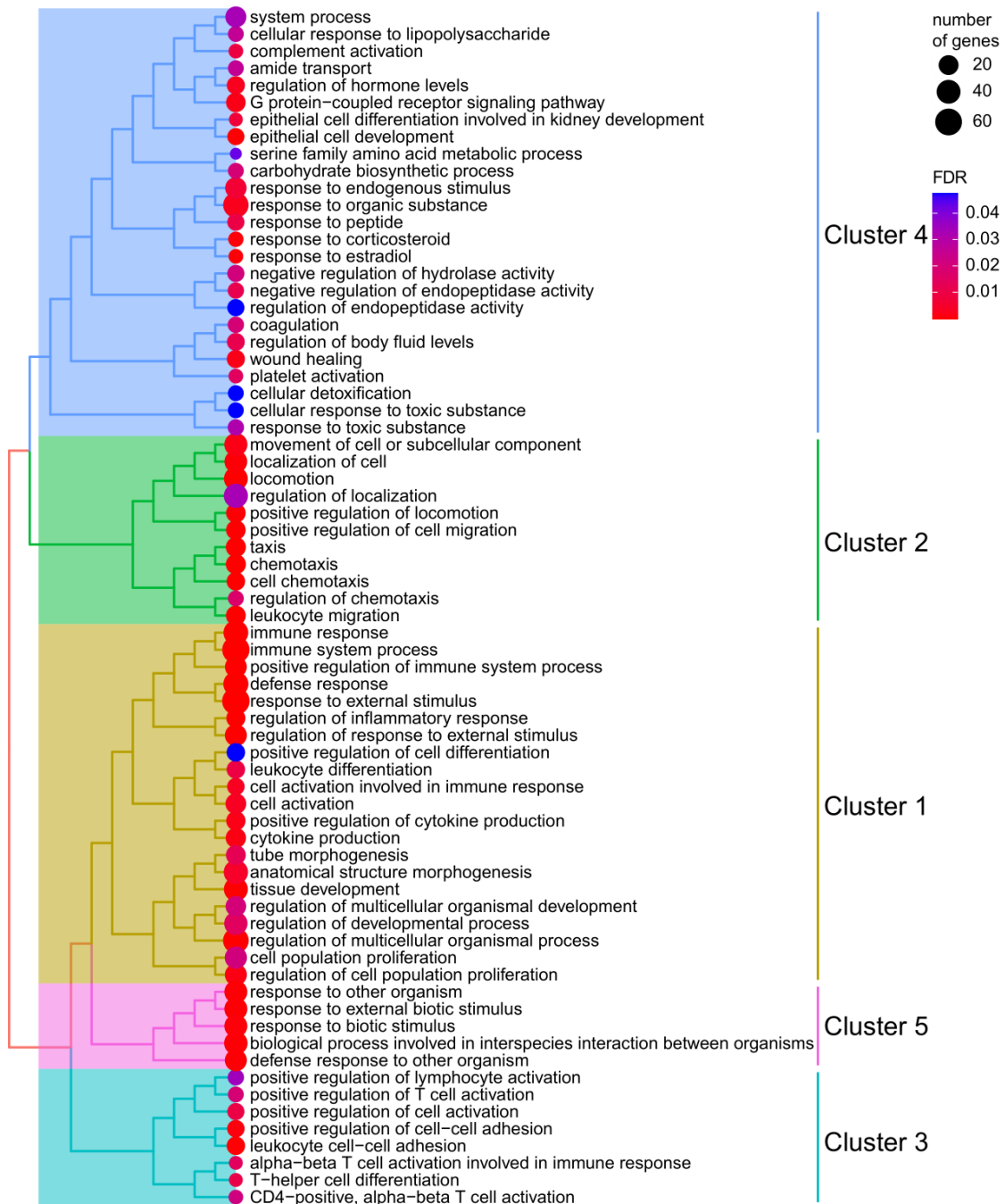

**Figure S1.** DMEG BP-GO treeplot.

Supplement: Supplementary file 1 [file ijms-24-10369-s001.zip › Figure S1 DMEG BP-GO treeplot..pdf]
